# Agentic artificial intelligence in radiology workflow: from image interpretation to report quality control

**DOI:** 10.3389/fmed.2026.1927284

**Published:** 2026-09-01

**Authors:** Wei Yi, Yajuan Chen, Xiaomeng Feng, Tikao Xia

**Affiliations:** 1 Department of Radiology, The Third People’s Hospital of Yunnan Province, Kunming, China; 2 Department of Plastic Surgery, The Second Affiliated Hospital of Kunming Medical University, Kunming, China

**Keywords:** agentic artificial intelligence, foundation models, large language models, multi-agent systems, quality control, radiology workflow, report generation

## Abstract

Radiology AI has grown past the single-purpose detector. The newest systems, built around large language models (LLMs), chain together the steps a radiologist actually works through: triaging the worklist, retrieving prior imaging studies, processing images, drafting a structured report, checking for mistakes. When these modules are coordinated through an orchestration layer, they form a multi-agent system capable of managing multiple stages of the radiology workflow—software that handles stretches of the radiology pipeline with less human input at each stage. This review maps the evidence behind that shift, drawing on PubMed-indexed studies from 2023 to 2026. We begin with convolutional neural networks and foundation models, then follow the emergence of AI agents that observe, plan, and act inside clinical environments. We examine multi-agent architectures—specialized agents for image analysis, report drafting, error detection, and decision support—and ask what they actually deliver. So far, the data tell a consistent story: multi-agent cross-verification drives hallucination rates down; intelligent worklist triage cuts report turnaround time by up to 43.7% in some settings; GPT-4 catches 82.7% of report errors, matching human readers. But nearly all of this evidence comes from single-center, retrospective studies on curated data. Every systematic review reaches the same conclusion: the technology works in the lab and has not been proven in the clinic. We also discuss compound opacity—how layered agent interactions make decisions harder to trace—alongside poor reporting standards and a regulatory framework that was not designed for generative, continuously-adaptive software. Agentic AI, including multi-agent architectures, could reshape how radiology departments operate, but the field needs prospective, multi-center trials with standardized endpoints before claiming it already has.

## 1 Introduction

Radiology buys more FDA-cleared AI software than any other medical specialty. By 2025, over 530 algorithms—about 76% of all cleared AI/ML devices—were listed for radiology, covering reconstruction, detection, segmentation, and worklist management (1, 2). Radiologists are not adopting AI out of curiosity. Imaging volumes keep rising while staffing does not, and the error rate in radiology reports has barely budged from 3 to 5% across decades of quality improvement work (3).

The first generation of radiology AI was narrow. CNNs trained to spot lung nodules or flag intracranial hemorrhages, each model doing one thing and waiting for a radiologist to trigger it (4, 5). These tools were accurate on benchmark datasets, but they sat outside the workflow. Someone opened an image, the model produced a number, and the radiologist decided whether to believe it. The sequence of steps that makes up a real radiology shift—triage, protocol check, retrieval of priors, interpretation, reporting, communication—remained human labor.

LLMs and vision-language foundation models broke that mold. A single model could process text, analyze images, and reason across both modalities. The next step was an agent: software that not only analyzes but decides what to do next, acts, checks the result, and adjusts. Faghani et al. call this the plan-action-observation cycle (6). Dietrich draws a simpler distinction—a tool waits for a command; an agent works through a queue on its own (7). When several such agents are wired together—one retrieving prior imaging studies, another analyzing images, a third drafting the report, a fourth proofreading—you get a multi-agent system (MAS) that can, in principle, run a substantial portion of the radiology pipeline (8, 9).

In 2025, more papers appeared on healthcare AI agents than in all previous years combined (10). However, the volume of publications does not constitute clinical evidence. Systematic reviews consistently identify a recurring pattern: studies are predominantly single-center, retrospective, and conducted on synthetic or curated datasets. Notably, it is the underlying machine learning components (e.g., LLMs and vision models) within these agentic systems that are trained and evaluated on such data, rather than the agentic architectures themselves, which are assembled from pre-trained models, external tools, memory, and orchestration mechanisms (11, 12). This review has three goals: to map the technical trajectory from task-specific AI to multi-agent architectures, to assess what the published evidence actually shows about image interpretation, report generation, and quality control, and to identify the gaps that still block clinical use.

## 2 From CNNs to foundation models to agents

### 2.1 Task-specific deep learning in radiology

For most of the past decade, radiology AI meant CNNs: U-Net for segmentation, YOLO and Faster R-CNN for detection, DenseNet and EfficientNet for classification (4, 13). A 2023 survey of CNN-based cancer detection across seven organ systems reported AUCs above 0.90 in controlled datasets (4). Momin et al. reviewed 38 studies of deep learning-driven worklist triage and found that actively reordering the reading queue cut report turnaround time by 43.7%, vs. 7.6% for systems that merely sent a notification (*p* < 0.01) (14). That is a real operational gain, not a benchmark number. Among CNN-based segmentation tools, total segmentator (15), which is built upon the U-Net framework, has emerged as a widely adopted solution for automated multi-organ segmentation, further illustrating the clinical utility of CNN architectures in radiology workflow applications.

But each of those models did exactly one thing. A pulmonary embolism detector could not generate a report. A laterality checker could not retrieve priors. Radiology, as a workflow, demands all of these tasks woven together, and single-purpose models were not built for that.

### 2.2 The foundation model paradigm

Foundation models (FMs) are large networks pretrained on broad data and adaptable to many downstream tasks, a genuine break from training one model per function. In radiology, three categories dominate: vision-only models for segmentation and anatomical parsing (MedSAM); vision-language models that link images to text (MedCLIP-SAM, BioMedCLIP, CheXzero), and LLMs adapted for clinical text (Med-PaLM 2, GPT-4 with radiology prompting) (16, 17).

Akinci D’Antonoli et al. reviewed FM applications across image interpretation, report generation, synthetic data, and decision support (16). Their review noted that only one FM-based product, Aidoc’s CARE1™, had FDA clearance as of 2025. Bian et al. compared task-specific models against FMs and found the real advantage was zero-shot and few-shot generalization—getting reasonable performance on new tasks without new labels—but domain gaps between natural images and medical images persisted (17). Noh and Lee reviewed 63 FM-based segmentation studies and found competitive 2D performance, though 3D CT and MRI segmentation still struggled with blurred boundaries and sparse annotated data (18).

### 2.3 The emergence of agentic AI

A foundation model does not become an agent merely by performing an action. Modern agentic AI systems are composite architectures that integrate multiple interdependent components: (i) a foundation model or LLM serving as the reasoning engine that interprets instructions and generates plans; (ii) tools (e.g., Python functions, APIs, software interfaces) that enable interaction with digital or physical environments; (iii) system instructions or policies that define the system’s behavior and constraints, and (iv) memory mechanisms (short-term and/or long-term) that maintain context across interactions. Faghani et al. (6) define the core operational loop as plan-action-observation: the agent perceives the environment, formulates a goal, executes an action, checks the result, and iterates. Within this architecture, the foundation model is one component of the broader agentic framework—supplying reasoning, language understanding, and visual processing capabilities—while the orchestration layer determines the sequence of actions: what to do next, which tools to invoke, and what information to retain in memory. Dietrich (7) framed this as a move from passive, user-triggered tools to systems that independently scan queues, prioritize by urgency, retrieve patient context, and act on it. He directly highlighted the gap between concept and deployment: most agentic systems are still in simulation, on retrospective data, inside one institution. PACS integration, regulatory uncertainty, and the lack of agreed evaluation protocols sit between the prototype and the clinic.

## 3 Multi-agent architectures in radiology

### 3.1 Design principles and orchestration patterns

Multi-agent systems break a clinical workflow into subtasks and assign each to a specialized agent, with an orchestration layer managing task allocation, inter-agent communication, and output synthesis. Three patterns appear in the literature: a supervisor agent that delegates and synthesizes (36% of clinical MAS studies); sequential pipelines where each agent hands output to the next (45%), and voting schemes where multiple agents tackle the same task and results are aggregated (9%) (10).

Salehi et al. [(8), Bioengineering] published a comprehensive review of agentic AI in radiology, covering multi-agent role-based systems, retrieval-augmented generation (RAG), and uncertainty quantification as strategies for reducing hallucination. A complementary review by Tzanis et al. [(19), Diagnostic and Interventional Imaging] provides a broader perspective encompassing fundamental principles, privacy, regulatory frameworks, and sustainability considerations in agentic AI for medical imaging. Single-agent radiology LLMs produced hallucinations in 8–15% of outputs. Multi-agent cross-verification plus RAG brought that close to zero in controlled experiments. However, those experiments used synthetic or curated test sets. Whether the same performance metrics generalize across institutions, scanners, or patient populations has not been empirically evaluated.

### 3.2 Representative multi-agent radiology systems

The Mayo Clinic group laid out a 9-agent trauma radiology workflow: protocol selection, triage, image enhancement, critical finding detection, structured reporting, and clinician communication (6). Each agent had a defined scope. The triage agent monitored the PACS worklist for new trauma CTs. The protocol agent confirmed the right scan was ordered. The detection agent detected hemorrhages, mass effect, fractures. The reporting agent produced a structured preliminary read. The communication agent escalated critical results. It is a coherent design, but it was a proof-of-concept. No prospective validation (Table 1).

**Table 1:** Representative multi-agent AI systems for radiology workflow.

System	Architecture/Agents	Task	Key finding
Mayo clinic trauma workflow [Faghani et al. (6)]	9-agent pipeline: protocol selection, triage, image enhancement, critical finding detection, structured reporting, clinician communication	Trauma radiology workflow automation	Sequential agent handoffs with decreasing human input at each stage (6)
MedXpert-CAD [MICCAI 2025 Workshop (20)]	4 sub-agents: supervisor, web search, chest X-ray expert, lumbar spine stenosis expert; powered by GPT-4o	Chest radiograph and lumbar spine MRI interpretation	90–96% task completion rate; multimodal multi-agent system with MCP-driven agentic workflows (20)
Medical AI consensus [NeurIPS (22)]	10 specialized agents with multi-agent reinforcement learning; radiologist-in-the-loop feedback	Radiology report generation and evaluation	Multi-agent consensus outperforms single-agent baselines (22)
Multi-agent RAG system [Kim et al. (21)]	Multi-agent retrieval-augmented generation pipeline bridging clinical narratives and ACR appropriateness guidelines	Medical imaging decision support	Clinical narratives successfully matched to ACR appropriateness criteria via multi-agent retrieval (21)
Concept bottleneck multi-agent RAG [ECIR (23)]	Concept bottleneck models combined with multi-agent RAG for interpretable report generation	Interpretable report generation	Improved interpretability through concept-level reasoning in multi-agent framework (23)
9-agent role-based system [Salehi et al. (9)]	Role-based multi-agent architecture with RAG and uncertainty quantification	General radiology workflow	Systematic review of multi-agent ethics in radiology; identified compound opacity as key concern (9)
RadFabric [Chen et al. (24)]	14 specialized open-source CXR analytics models + 2 VLMs; localized reasoning via agentic orchestration	Chest X-ray interpretation with step-by-step explainable diagnosis	Agentic orchestration of 14 specialized CXR analytics models and two vision-language models enables explainable, step-by-step diagnosis through localized reasoning (24)
ReclAIm [Tzanis & Klontzas (25)]	Master agent + 3 task-specific agents: image classification, performance comparison, fine-tuning	Performance monitoring and auto-fine-tuning of medical image classification models	Detected degradation in 8/18 models (up to 40.6% drop); auto-fine-tuning restored performance to within ±2% of baseline values (25)

MedXpert-CAD (20) used four sub-agents—supervisor, web search, chest X-ray expert, lumbar spine stenosis expert—powered by GPT-4o, and reported 90–96% task completion on chest radiographs and lumbar spine MRI. A separate multi-agent RAG system that maps clinical narratives to ACR Appropriateness Criteria achieved 81% exact match accuracy and 93.9% top-10 recall (21). These are solid engineering results, but they are demonstrations on curated tasks.

At NeurIPS (22), a Medical AI Consensus framework deployed 10 specialized agents with multi-agent reinforcement learning for report generation and evaluation, using radiologist feedback in the loop. At ECIR (23), a multi-agent RAG system combined concept bottleneck models with REACT agents for interpretable chest X-ray reports. Beyond these conference presentations, several recent high-impact publications have advanced the state of the art. Chen et al. [(24), npj Digital Medicine] introduced RadFabric, an agentic AI system that orchestrates 14 specialized open-source chest X-ray analytics models and two vision-language models to produce explainable, step-by-step radiology diagnoses through localized reasoning. Tzanis and Klontzas [(25), Radiology: Artificial Intelligence] developed ReclAIm, a multi-agent LLM-based framework for automated monitoring, detection, and correction of performance decline in medical image classification models. ReclAIm employs a master agent coordinating specialized agents for image classification, performance comparison, and autonomous fine-tuning, successfully detecting performance degradation in 8 of 18 tested models and restoring performance to within ±2% of baseline values through automated fine-tuning workflows. Furthermore, Ferber et al. [(26), Nature] introduced MIRA (Medical Intelligence for Reasoning and Action), a generalist medical AI agent designed for autonomous emergency department workflows. Powered by GPT-4o and operating in a sandboxed EHR environment, MIRA navigates over 85,000 clinical decision options across eight emergency department diagnoses. While not exclusively a radiology system, MIRA autonomously orders and interprets imaging studies—including CT and MRI—as part of its diagnostic workup, achieving 88.9% diagnostic accuracy. Its relevance to radiology lies in demonstrating how agentic AI can integrate imaging interpretation within a broader clinical reasoning pipeline. Recent studies have introduced increasingly sophisticated agentic architectures. Prospective, multicenter clinical validation of these representative systems remains absent.

### 3.3 Systematic review evidence

The systematic reviews agree on this point. Gorenshtein et al. (10) found 20 peer-reviewed studies of AI agents in clinical medicine, all from 2024 to 2025. Only 30% had low risk of bias. 65% used synthetic or curated data. Zero were multi-center prospective trials. Collaco et al. (11) identified seven qualifying studies across all of healthcare, with multi-agent architectures the most common pattern, but only one study had been deployed with real patients. Ode et al. (27) reviewed 32 healthcare MAS studies and found over 60% were proof-of-concept, with seven discussing ethics or legal implications at any depth. Four independent reviews, one conclusion: the technology shows promise in the lab and has not been validated in the clinic.

## 4 Image interpretation: CNNs to agentic vision-language models

### 4.1 Conventional deep learning performance

Chest X-ray interpretation has been the most heavily studied application, with deep learning now covering classification, detection, segmentation, and automated report generation (28). A 2024 systematic review of 311 object detection papers found YOLO variants and the R-CNN family dominant across CT, MRI, pathology, endoscopy, and radiography, though the reviewers noted that most training sets are not public (13).

Even high-performing models have had limited clinical impact because integration is hard. Korfiatis et al. (2) listed the bottlenecks: computational infrastructure, PACS/EHR interoperability, post-deployment monitoring, governance. Jiang et al. (29) added real-world validation, user training, and continuous monitoring to the list. Accuracy on a benchmark is the beginning of the problem, not the end.

### 4.2 Foundation models for image interpretation

Foundation models change the economics of image interpretation because one pretrained model handles multiple tasks. Bian et al. showed that vision-language FMs like BioMedCLIP and CheXzero approach the zero-shot classification accuracy of fully supervised CNNs on chest X-ray pathology without any task-specific training data (17). MedSAM, trained on 1.5 million image-mask pairs, generalizes across ultrasound, CT, MRI, and dermatoscopy images, though 3D volumes remain difficult (18).

An agentic wrapper around a foundation model adds a decision layer: which images to process, in what order, with which context. Faghani et al. described a trauma case where the agent detected an acute intracranial hemorrhage, pulled the patient’s coagulation labs, noted an elevated INR, and folded that into a structured report with a neurosurgery recommendation (6). A single-purpose detector cannot do that.

### 4.3 Evidence gaps

The systematic review of agentic AI in neuroradiology (2026, Journal of Imaging Informatics in Medicine) screened 230 records. Nine met the criteria for true agentic AI: autonomous observation, planning, and action. About 30% of the screened papers had been incorrectly classified as agentic AI despite representing conventional deep learning models. No study included a safety assessment. One randomized trial, INSPIRE, was identified. The authors’ summary: ‘technical promise with limited clinical evidence’ (12).

## 5 Report generation: LLMs as reasoning engines within agentic systems for structured reporting

### 5.1 The case for automated report generation

Although LLMs are not themselves agents, they serve as the core reasoning engine within the majority of modern agentic radiology systems. In an agentic reporting workflow, the LLM does not operate in isolation; it interacts with tools for data retrieval, image analysis modules, template engines, and verification agents that together constitute the reporting pipeline. This section reviews the evidence for LLM-based report generation, with attention to how these models function as components within broader agentic architectures. Radiology reporting is still mostly manual dictation. A radiologist in a busy practice may produce over 100 reports per shift. The error rate stays around 3–5%: laterality mistakes, omissions, insertions, interpretive misses (3). Structured reporting, using templates aligned with ACR Reporting and Data Systems, improves completeness and makes data extractable for research and audit. But it adds time, and that is why voluntary adoption has been slow. An LLM that drafts the structured report, leaving the radiologist to edit and attest, could reduce both the cognitive load and the turnaround time (Table 2).

**Table 2:** LLM-based radiology report generation and quality control: representative studies.

Study	Model/Approach	Task	Performance
Zhang et al. (31)	Purpose-trained LLM: 20 GB pretraining + 1.5 GB fine-tuning on 800 reports	Impression generation from findings (CT, MRI, X-ray)	F1 0.772; higher completeness, correctness, brevity than GPT-4 and radiologist reports (31)
Chen et al. (32)	Curriculum learning: disease labels → entity-relation triples → full reports	Chest X-ray report generation	State-of-the-art BLEU, ROUGE-L, METEOR scores on MIMIC-CXR and IU X-ray (32)
Busch et al. (30)	Systematic review of 10 LLM studies for structured reporting	Multilingual structured reporting	6/10 studies: multilingual structured reporting feasible; 0/10 in real workflows (30)
Artsi et al. (33)	Systematic review of 9 LLM reporting studies	Report generation quality	Hallucinations 44%, misdiagnoses 55%, missing details 66%; 0% external validation (33)
Gertz et al. (3, 36)	GPT-4 for radiology report error detection	Error detection in 200 reports	Sensitivity 82%, specificity 92% for clinically significant errors (3)
Kathait et al. (37)	ATARI (generative AI) vs. commercial NLP tool	Laterality error detection (898 reports)	ATARI: Text query: 97.4% accuracy; Vision + text: 98.3% accuracy; commercial tool: 36% accuracy, 64% false positives (37)
Salam et al. (38)	Open-source LLMs: Llama 3-70B, Mixtral 8 × 22B	Error detection benchmark	Open-source LLMs reached 85–95% of GPT-4 performance; no data leaving hospital (38)
Multi-Pass Agentic Pipeline [JMIR (41)]	Extract → detect → verify agentic pipeline	Report error detection with PPV optimization	PPV ↑ from 6.3 to 15.9%; inference cost ↓ 42.7% vs. single-pass (41)

### 5.2 LLMs for report generation: current evidence

Busch et al. [(30), European Radiology] published the first narrative review of LLMs for structured reporting, covering 10 studies. Six showed that multilingual structured reporting was feasible. The review identified four use cases: structuring free-text into templates, translation and patient-facing summarization, clinical information extraction, and data mining. The authors flagged opaque training data, inconsistent model version reporting, and the regulatory vacuum for LLM-generated clinical documents as unresolved problems. From an agentic perspective, these LLM-based reporting functions represent the reasoning and text-generation component of a larger system that may additionally include image analysis agents, fact-verification modules, and template-enforcement tools. The integration of LLM-generated reports into a multi-agent quality-control pipeline—where a separate agent cross-checks the report against imaging findings—is discussed in Section 6.

Zhang et al. [(31), Radiology] built a purpose-trained LLM: 20 GB of pretraining on medical and general text, 1.5 GB of fine-tuning on 800 reports with paired instructions. On 3,988 patients across CT, MRI, radiography, and mammography, the model produced impressions from findings with a median F1 of 0.772 (IQR 0.578–0.957). An expert panel rated the output 5/5 across terminology, coherence, correctness, comprehensiveness, and safety. The panel could not reliably distinguish the model’s impressions from radiologist-authored text.

Chen et al. [(32), IEEE TMI] introduced a curriculum-learning approach: train on disease labels first, then entity-relation triples, then full reports. This three-stage supervision produced state-of-the-art fluency and clinical accuracy. The insight is that end-to-end training on whole reports washes out the clinically loaded phrases, like ‘cannot exclude pulmonary embolism,’ that carry most of the diagnostic weight.

### 5.3 Limitations of current report generation systems

Artsi et al. (33) systematically reviewed 9 LLM reporting studies and found hallucinations in 44%, misdiagnoses in 55%, and missing clinical details in 66%. The metrics used to evaluate these systems are part of the problem. BLEU, ROUGE, and METEOR reward surface-level similarity to reference reports and miss clinically dangerous errors. Zhu et al. (34) showed that LLM-based evaluators using in-context learning and chain-of-thought reasoning correlated far better with expert judgment (*r* = 0.64 for regressed GPT-4) than any conventional NLG metric. Yuan et al. (35) built NRRBench specifically to measure report cleanliness, radiological usefulness, and factual correctness, and their LLM-based metrics tracked error rates far more closely than BLEU, ROUGE, or METEOR. Conventional evaluation metrics may inadequately capture clinically significant report errors. When considered within an agentic framework, these limitations highlight the need for complementary agents—such as dedicated fact-verification modules and image-text alignment checkers—that can detect and flag errors an LLM alone may introduce, thereby strengthening the overall reliability of the reporting pipeline (Table 3).

**Table 3:** Key challenges, current evidence, and proposed directions for agentic AI in radiology.

Challenge	Description	Current evidence	Proposed direction
Hallucination [Salehi et al. (8)]	LLMs fabricate measurements, reference non-existent priors, invent pathological findings	Multiple documented hallucination modes in radiology LLM outputs; no standardized detection framework (8)	Retrieval-augmented generation (RAG), structured output constraints, human-in-the-loop verification (8, 23)
Compound opacity [Salehi et al. (9)]	Multiple opaque agents interacting create emergent uncertainty beyond the sum of individual opacities	Coined and defined in radiology multi-agent context; no quantitative measurement framework exists (9)	Agent-level explainability, interaction logging, uncertainty propagation tracking (9)
Insufficient reporting [Trägårdh et al. (43)]	246 LLM-radiology studies audited against MI-CLEAR-LLM and TRIPOD-LLM checklists	27.6% reported model version; 33.7% reported prompt; 42.7% reported temperature; 7.3% externally validated (43)	Extend MI-CLEAR-LLM and TRIPOD-LLM to multi-step agentic workflows; journal enforcement (43)
Regulatory uncertainty [Gowda et al. (44); FDA (1)]	FDA 510(k)/*De Novo* designed for locked software; LLM-based agentic systems may exhibit behavior that changes with prompt modifications without retraining	>530 radiologic AI algorithms cleared as locked Class II; no clearance pathway for self-modifying agents (1, 44)	Trilaminar regulatory framework: locked → semi-adaptive → fully adaptive tiers (44)
Validation gap [Salehi et al. (12); Gorenshtein et al. (10)]	Systematic reviews consistently find near-zero prospective validation, external testing, or safety assessment	Neuroradiology: 230 records → 9 agentic, 0 safety assessments, 1 RCT (12); Clinical: 20 studies, 30% low bias risk (10)	Prospective multi-center trials; standardized safety endpoints; TRIPOD-Agentic extension (10, 12)
Workflow integration [Korfiatis et al. (2)]	Computational infrastructure, PACS/EHR interoperability, post-deployment monitoring remain unsolved	Bottlenecks catalogued but no deployed multi-agent system has demonstrated end-to-end clinical integration (2)	FHIR/DICOM-web standards; edge-cloud hybrid deployment; continuous monitoring dashboards (2, 29)
Cost–benefit uncertainty [Dietrich (7)]	Multi-agent systems incur higher computational cost; clinical benefit vs. cost not quantified	Single editorial calling for cost-effectiveness analysis; no published cost–benefit data for agentic radiology AI (7)	Health-economic modeling; incremental benefit over single-agent and no-AI baselines (7)

### 5.4 From standalone LLMs to agentic reporting pipelines

The trajectory from standalone LLMs to agentic reporting pipelines represents a fundamental architectural shift. Early work, reviewed in Sections 5.1–5.3, treated LLMs as isolated text generators: a model received findings and produced an impression. In contrast, an agentic reporting pipeline embeds the LLM within a broader architecture that includes image analysis agents for visual grounding, retrieval agents for accessing prior studies and clinical context, template-enforcement agents for structured output compliance, and verification agents that cross-check report content against imaging findings (Section 6). This decomposition transforms report generation from a single inference call into a coordinated, multi-step workflow in which each agent contributes a specialized capability. The evidence reviewed in Sections 5.2–5.3 demonstrates that LLMs alone can produce fluent reports but remain prone to hallucinations and omissions; the agentic pattern addresses these limitations by distributing verification and context retrieval across specialized modules. Future agentic reporting systems are likely to incorporate memory mechanisms for longitudinal patient context, enabling each new report to be generated with awareness of the patient’s full imaging history rather than treating each study in isolation.

## 6 Report quality control: error detection and correction

### 6.1 LLMs for radiology report error detection

Error detection may be the most immediately usable application of LLMs in radiology, because it functions as a safety check rather than a replacement for radiologist judgment. Gertz et al. [(36), Radiology] tested GPT-4 on 200 reports seeded with common errors: omissions, insertions, spelling mistakes, side confusions. GPT-4 caught 82.7% of errors. Senior radiologists caught 89.3%, attendings and residents 80.0%. The difference was speed: GPT-4 took 3.5 s per report at $0.03; the fastest human reader took 25.1 s at $0.42. For high-volume screening, the economic implications warrant careful consideration.

Kathait et al. [(37), JACR] compared a generative AI prototype, ATARI, against a commercial NLP tool for laterality errors. The commercial tool managed 36% accuracy with a 64% false-positive rate on 898 flagged reports. ATARI with text queries hit 97.4%. Adding image queries pushed it to 98.3%. A system that checks the image against the words in the report catches errors that are invisible to text-only tools.

### 6.2 Open-source and fine-tuned approaches

Sending clinical reports to commercial cloud APIs raises legitimate data-security and privacy concerns among hospital data-protection officers. Salam et al. [(38), European Radiology] benchmarked open-source models (Llama 3-70B, Mixtral 8 × 22B) against GPT-4 and GPT-4o on 120 reports with 397 inserted errors. GPT-4o led at 88% detection, GPT-4 at 83%, Llama 3-70B at 79%, Mixtral at 73%. The open-source models ran faster (6 vs. 13 s per report) and stayed inside the hospital firewall. Numerical errors were the easiest to catch (88%); interpretive errors were the hardest (70%). Sun and Peng et al. [(39), Radiology] showed that fine-tuning LLMs on MIMIC-CXR plus synthetic reports beat baseline GPT-4 and BiomedBERT. The pattern is consistent: domain-specific fine-tuning on real clinical reports outperforms larger, general-purpose models.

### 6.3 From single-pass detection to multi-agent quality control

Single-pass LLM error detection generates too many false positives on real-world data because actual error rates are low. Weng et al. [(40), JMIR Formative Research] tackled laterality errors in combined reports, where the error rate was 1.47% vs. 0.57% in simple reports, using a rule-based plus GPT-4o ensemble. They found a large performance drop between balanced synthetic test sets and real clinical data.

A multi-pass agentic pipeline—extract, then detect, then verify—lifted the positive predictive value of LLM error detection from 6.3 to 15.9% and cut inference cost by 42.7% (41). That is the agentic pattern in miniature: a single call is noisy, a chain of specialized checks is sharper. At scale, Pan et al. (42) deployed RadCLARE, a BERT-based engine trained on 1.4 million Chinese radiology reports. After deployment, the semantic error rate fell from 4.19 to 0.85%, and 95.7% of radiologists said they were satisfied. This is one of a handful of studies showing real-world impact rather than benchmark performance.

## 7 Workflow orchestration: triage, prioritization, integration

### 7.1 Intelligent worklist management

Worklist triage provides an important precursor and foundation for agentic workflow orchestration in radiology. Momin et al. [(14), European Radiology] systematically reviewed 38 studies (1,38,423 images) and reported mean turnaround-time reductions of 12.3 min for pulmonary embolism, 20.5 for stroke, 4.3 for intracranial hemorrhage, and 29.7 for chest diseases. The mechanism mattered: algorithms that actively reordered the worklist cut turnaround time by 43.7%; passive notification widgets cut it by 7.6% (*p* < 0.01). Outpatient and high-prevalence settings gained the most. Two open questions: how to handle false negatives when the algorithm deprioritizes a positive case, and how to prioritize across multiple suspected conditions simultaneously.

### 7.2 Comprehensive workflow integration

Triage is one node. An agentic system could also verify the imaging protocol, cross-check contrast allergies, retrieve priors, select key images, populate measurements, draft the structured report, communicate critical results, and track follow-up recommendations. Korfiatis et al. (2) mapped the infrastructure this would require—orchestration engines, FHIR and DICOMweb interoperability, continuous monitoring—and stressed that governance, stakeholder buy-in, and change management matter as much as the algorithms. The Mayo 9-agent trauma workflow (6) and the multi-agent RAG system for ACR criteria (21) are early prototypes of full orchestration. Neither has been prospectively validated. Across this literature, one pattern dominates: prototypes function in controlled settings while prospective clinical deployments are absent (7, 8, 12).

## 8 Challenges and limitations

### 8.1 Hallucination and factual reliability

LLMs may generate factually incorrect or hallucinated outputs. In radiology, a hallucination can be a fabricated measurement, a reference to a prior study that does not exist, or an invented pathological finding. Salehi et al. (8) documented baseline rates of 8–15% in single-agent systems. Multi-agent cross-verification, RAG, and uncertainty quantification cut those rates in the lab, However, these approaches have not been evaluated under clinically relevant distributional shifts: different institutions, different scanners, different patient populations. Until such experiments are conducted under clinically representative conditions, the safety estimates remain provisional.

### 8.2 Compound opacity

Salehi et al. [(9), Bioengineering] coined ‘compound opacity’ for what happens when multiple opaque agents interact. With one model, a radiologist can look at input and output and form a judgment. With nine agents exchanging state information, tracing a decision becomes much harder. The authors call this the autonomy–transparency paradox: the more you let the system do, the less you can see inside it. They argue that multi-agent deployment needs audit trails, per-agent explanations, and mandatory human review for high-stakes decisions. Whether that is practical at scale is an open question.

### 8.3 Insufficient reporting standards

A systematic review in Insights into Imaging (2026) checked 246 LLM-radiology studies (November 2022 to December 2024) against the MI-CLEAR-LLM and TRIPOD-LLM checklists (43). Only 27.6% reported the exact model version. Only 35.8% gave the access date. Only 41.1% provided full prompts. Only 16.7% reported the temperature parameter. These gaps persisted after TRIPOD-LLM was published in July 2024, which suggests that voluntary checklists are not enough. Journals may need to mandate them.

### 8.4 Regulatory uncertainty

The FDA has cleared over 530 radiologic AI algorithms, mostly as Class II devices under 510(k) or *De Novo* (1). Those pathways were designed for locked, task-specific software. An LLM-based agent—continuously adaptive, general-purpose, behavior-shifting with prompt changes—does not fit that mold. Zhang et al. (1) walked through the predetermined change control plan (PCCP) framework for adaptive algorithms and noted it applies to a small fraction of cleared devices and was never meant for generative AI. Gowda et al. (44) proposed a three-layer governance model: federal regulation as the floor, institutional policies in the middle, industry self-regulation on top. The core tension is that a generative model’s output can change without retraining, just from a different prompt, and existing regulatory frameworks have no mechanism for that.

### 8.5 The validation gap

Every systematic review of this literature arrives at the same place. In neuroradiology: 230 records, 9 met agentic criteria, zero included safety assessments, one RCT (12). In clinical medicine: 20 AI agent studies, zero multi-center trials (10). In healthcare MAS: 32 studies, over 60% proof-of-concept (27). The field keeps producing strong technical results in curated settings and has yet to produce evidence of clinical benefit in real-world settings. That gap is the central fact about this literature.

## 9 Future directions

### 9.1 Priorities for clinical translation

Four priorities should guide future clinical translation. First, evaluation standards for agentic systems—extending TRIPOD-LLM and MI-CLEAR-LLM to multi-step workflows—need to be adopted and enforced by journals and regulators (43). Second, prospective multi-center trials with clinical endpoints (diagnostic accuracy, turnaround time, error detection, radiologist efficiency, patient outcomes) are a prerequisite for regulatory clearance and for convincing anyone outside the lab that these systems work (10, 12). Third, multi-agent explainability and audit mechanisms need to be built into the architecture, not bolted on afterward, to address compound opacity (9). Fourth, regulators need a framework for continuously adaptive LLM-based systems that does not demand full re-validation after every prompt tweak (1, 44). Each of these is a multi-year project.

### 9.2 Emerging research directions

Several lines of work deserve attention. Memory-augmented agents that maintain context across longitudinal imaging could enable something closer to personalized radiology—reading each study against the patient’s full imaging history rather than treating it as a first encounter. Federated multi-agent learning could let institutions collaborate on models without moving protected health data, though the practical barriers are substantial. Human-agent teaming models that divide work based on case complexity and uncertainty—simple cases to the machine for triage, ambiguous cases to the radiologist with the machine’s notes—could optimize the division of labor without pretending the machine can handle everything. Safety-first agent architectures with mandatory human review for high-risk findings and formal verification of critical paths could address the autonomy–transparency tension directly, assuming verification techniques can scale to the complexity of real clinical reasoning.

## 10 Conclusion

Agentic AI, including multi-agent architectures, represents a substantive shift in the capabilities of radiology software. Instead of a collection of single-purpose detectors, these systems chain together triage, retrieval, interpretation, reporting, and quality control into something closer to a continuous pipeline. Individual components work: CNNs and foundation models interpret images at or near expert level; LLMs generate reports that panels cannot distinguish from human drafts; GPT-4 catches errors as well as attending radiologists; multi-agent architectures drive hallucination rates down through cross-verification. But the clinical evidence is not just thin—it is almost nonexistent. Across systematic reviews, fewer than 10 agentic AI studies meet rigorous inclusion criteria. None demonstrates prospective clinical benefit in a multi-center design. The literature shows these systems can be built. It does not show they help patients. Closing that gap will take trials designed to convince people outside the AI community—which means prospective, multi-center, with pre-registered endpoints, run on the kinds of heterogeneous real-world clinical data that radiology departments routinely generate.

## Glossary

ACR: American College of Radiology
AI: Artificial Intelligence
AUC: Area Under the Curve
BERT: Bidirectional Encoder Representations from Transformers
CAD: Computer-Aided Diagnosis
CNN: Convolutional Neural Network
CT: Computed Tomography
DL: Deep Learning
EHR: Electronic Health Record
FDA: U. S. Food and Drug Administration
FM: Foundation Model
GDPR: General Data Protection Regulation
HIPAA: Health Insurance Portability and Accountability Act
JACR: Journal of the American College of Radiology
LLM: Large Language Model
LVM: Large Vision Model
MAS: Multi-Agent System
MRI: Magnetic Resonance Imaging
NLG: Natural Language Generation
NLP: Natural Language Processing
PACS: Picture Archiving and Communication System
PCCP: Predetermined Change Control Plan
PPV: Positive Predictive Value
RADS: Reporting and Data Systems
RAG: Retrieval-Augmented Generation
RIS: Radiology Information System
RTAT: Report Turnaround Time
SaMD: Software as a Medical Device
SR: Structured Reporting
